# The Power of Musification: Sensor‐Based Music Feedback Improves Arm Swing in Parkinson's Disease

**DOI:** 10.1002/mdc3.13352

**Published:** 2021-10-22

**Authors:** Stefan Mainka, Arno Schroll, Elke Warmerdam, Florin Gandor, Walter Maetzler, Georg Ebersbach

**Affiliations:** ^1^ Movement Disorders Hospital, Kliniken Beelitz GmbH Beelitz‐Heilstätten Germany; ^2^ Department of Training and Movement Sciences Humboldt‐Universitat zu Berlin Berlin Germany; ^3^ Department of Neurology Christian‐Albrechts‐Universitat zu Kiel Medizinische Fakultat Kiel Germany; ^4^ Department of Neurology Otto‐von‐Guericke University Magdeburg Germany

**Keywords:** overground walking, arm swing, musification, rhythmic auditory stimulation, Parkinson's disease

## Abstract

**Background:**

Reduction of arm swing during gait is an early and common symptom in Parkinson's disease (PD). By using the technology of a mobile phone, acceleration of arm swing can be converted into a closed‐loop musical feedback (musification) to improve gait.

**Objectives:**

To assess arm swing in healthy subjects and the effects of musification on arm swing amplitude and other gait parameters in patients with PD.

**Methods:**

Gait kinematics were analyzed in 30 patients during a 320 m walk in 3 different conditions comprising (1) normal walking; (2) focused swinging of the more affected arm; and (3) with musification of arm swing provided by the iPhone application CuraSwing. The acceleration of arm swing was converted into musical feedback. Arm swing range of motion and further gait kinematics were analyzed. In addition, arm swing in patients was compared to 32 healthy subjects walking at normal, slow, and fast speeds.

**Results:**

Musification led to a large and bilateral increase of arm swing range of motion in patients. The increase was greater on the more affected side of the patient (+529.5% compared to baseline). In addition, symmetry of arm swing, sternum rotation, and stride length increased. With musical feedback patients with PD reached arm swing movements within or above the range of healthy subjects.

**Conclusions:**

Musification has an immediate effect on arm swing and other gait kinematics in PD. The results suggest that closed‐loop musical feedback is an effective technique to improve walking in patients with PD.

Arm swing (AS) is an essential component of human gait that is often ignored in clinical studies. Its precise role in motor control is still controversial.^1^ Balance and energy expenditure during gait are possible functions of AS. However, the clinical significance of AS is discussed critically.^1^, ^2^ In Parkinson's disease (PD), asymmetric degeneration of dopamine projections leads to a predominant disturbance of automatized motor routines whereas attention‐guided motor behavior is often less impaired.^3^ As a consequence, asymmetric reduction of habitual AS with reduced active retroversion of the arm on the more affected side of the body is frequently observed^4^, ^5^, ^6^ and can even occur in prodromal stages of the disease.^7^

AS is a potential target for motor training in PD for several reasons. First, there is a potential role of AS for gait stability and, in this sense, reduced AS has been associated with an increased risk of falls in PD.^8^ Second, reduced AS during gait appears to be less responsive to dopaminergic medication and deep brain stimulation than leg movements.^9^ Third, improving AS has the potential to affect other gait kinematics including stride and trunk rotation because upper and lower limb movements influence each other during locomotion.^1^

Sensory cueing, motivation, and movement awareness are key elements of gait rehabilitation in PD. In rhythmic auditory stimulation (RAS), an external rhythmic stimulus presented as a simple pulsed beat or music is matched first to the patient's cadence and then shaped to optimize the locomotor rhythm.^10^ RAS has been shown to improve gait kinematics of the lower limbs.^11^ Recent data also suggest a positive effect on AS.^12^ Music has the potential to enhance the effects of simple acoustic cues because of its motivating properties^13^, ^14^ and has been shown to be more effective than a metronome for audio‐motor entrainment in patients with PD.^15^ Sonification is a further therapeutic approach that uses acoustic stimuli to enhance motor function. In contrast to the feedforward stimulation and the entrainment of locomotion to musical rhythms in RAS, sonification serves as a cross‐modal biofeedback. Here, sensor‐based movement data are transformed into acoustic signals to provide online access to biomechanical information.^16^ The generation of audio signals from kinematic data relies on a systematic algorithm that is called mapping. This mapping is tailored to a specific target movement to optimize motor learning.^16^

Movement sonification aims to assist motor control by improving self‐awareness of movement execution, which is often disturbed in PD.^17^, ^18^, ^19^ At present, sonification has been used mainly in instrumented footwear to provide acoustic cues to optimize steps and walking patterns in PD.^16^ Thompson et al^20^ showed that vibratory acoustic feedback improves amplitude of AS in short distance walking at comfortable speed.

Analogous to the advantages of music over metronomic stimuli, it has been suggested that music might be a more powerful means for transmitting information than simple sonification.^21^ The term musification has been coined to describe the musical representation of data.^22^ Recently, a sensor‐based music feedback system has been shown to induce larger steps in patients with PD.^23^

In the present study, a smartphone worn on the wrist was used to capture kinematic sensor data of AS during gait in patients with PD. The CuraSwing application converted these sensor data into a music signal that varied with changes in AS amplitude (“musification” of movement). Our rationale was that this musical bio‐feedback will improve sensorimotor processing and aid to maintain the attentional focus on enlarged swinging of the more affected arm. The musical output followed the principles of RAS providing a constant pulse‐accented stimulation. We hypothesized that the combined effects of bio‐feedback and rhythmic entrainment provided by musification would exceed the improvement of AS elicited by focused attention alone.^24^ We further assumed that optimizing movements of the more affected side would exert effects on the kinematics of the contralateral arm and leg movements during gait. To reference AS in patients to physiological measures, we compared their kinematic data to those acquired from healthy subjects walking at different velocities.

## Methods

The study was registered at the German clinical trials register (DRKS00022051). We included patients diagnosed with PD according to United Kingdom (UK) Brain Bank criteria that met the following criteria: age 40 to 79 years, ability to walk safely without use of walking‐aids, reduced 1‐sided AS amplitude (≤25°) as determined during normal walking with APDM Mobility Lab (ML) system (version 2, APDM, Portland, USA), ability to perform testing without fatigue based on previous experience of the participant, no further gait disorder not related to PD, no troublesome dyskinesia in on‐state (corresponding to score 0 in UPDRS item IV.33), no camptocormia or Pisa‐syndrome >20°, no freezing of gait during straight walking episodes during the last 6 months, and no signs or history of cognitive impairment (corresponding to score 0 in UPDRS item I.1.).

We calculated the levodopa equivalent daily dose for each patient by using the conversion factors of Schade and colleagues.^25^ In cases with response fluctuations (n = 11) assessments were conducted in the medication ON state. The number of 30 PD participants for the musification experiment was based on preliminary clinical observations without formal power calculation. We recruited age‐matched healthy individuals with good walking ability for collecting reference data of physiological walking.

Participants were instrumented for gait analysis with 6 inertial measurement units (IMU; Mobility Lab, APDM, USA), comprising sensors at the fifths lumbar vertebrae (5LV) and sternum, and 1 sensor fixed to each metatarsus and wrist dorsally with a Velcro strap. The 4.8 × 1.3 × 3.6 cm IMUs weigh 22 g and measure with a sampling frequency of 128 Hz. Cadence, gait velocity, stride length, stride time (values taken from the right body side), and range of motion (ROM) in the transversal (obliquity) and sagittal (rotation) plane of the 5LV and the sternum were computed by APDM Mobility Lab (ML) system (version 2).^26^ We assessed stride time variability by calculating the coefficient of variation (CV) with the following formula: CV = (standard deviation/mean) × 100.

The algorithm by Warmerdam and colleagues^27^ was used to compute the AS parameters ROM, peak angular velocity (PAV) and regularity, which represents the similarity of neighboring swings based on the angular velocity. AS ROM values were used to calculate the non‐directional asymmetry indices (ndASI) with the formula,

$$\text{ndASI}=\mathrm{ABS}\left(\frac{L-R}{\max\left(L,R\right)}\right)\times 100,$$

where R and L represent right and left AS ROM mean values.^28^ Assessments for patients with PD comprised of the UPDRS III motor score as well as three 320 m walks along a corridor with 180° turns every 80 m. At first, subjects were asked to walk at their individual comfortable speed (baseline). For the second walk they were instructed to walk at the same speed with as much AS as possible on their more affected side (focused). This walk was carried out with the smartphone attached to the wrist of the more affected arm to control for the impact of the weight of the device and the pouch, which was 196 g in total. The third walk was carried out with additional musical feedback delivered via headphones (Sony MDR‐ZX110) from a smartphone (Iphone 6s, Apple, USA) attached to the wrist with a pouch and Velcro straps. For the musical feedback the app CuraSwing was used. The CuraSwing application was designed for iOS smartphones by a developer‐team including 2 of the authors (S.M. and G.E.) at the movement disorder clinic in Beelitz‐Heilstaetten, Germany (available without charge at the apple app‐store). This application uses the acceleration values of the frontal (z) and longitudinal (x) plane of the smartphone to generate the musical feedback. The sample rate of the sensors is 20 Hz. Absolute x‐ and z‐acceleration values are transformed into 1 parameter. The algorithm is based on the principles of an envelope follower, which results in a 1‐sided flattening of the underlying cyclic wave motion. Based on the assumption that subjects walk faster and slower according to the pre‐set musical tempo, the resulting parameter is adjusted according to the tempo of the music. Therefore, different swing cycle durations are evened out to get comparable musification feedback for equal AS amplitudes at different step frequencies. The generated output is assigned to 10 threshold values representing 10 stages of increasing AS.

**FIG. 1:**
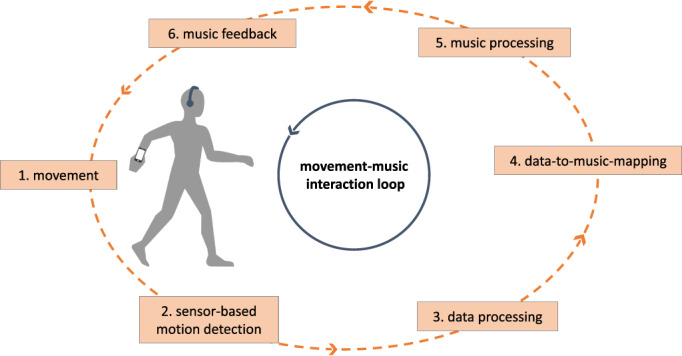
Functionality of the app CuraSwing.

The structure of the music follows the principles of RAS by providing a clear rhythmic structure with an even meter, a salient beat and a stable tempo. The musical feedback is generated by a software patch (PureData) from 6 audio layers representing distinct components of the musical composition (melody, harmonic accompaniment, snare, hi‐hat, kick drum and bass). The layers are filled with different audio files (.wav format), each of them being 32 bars long in the genre electro pop. The mix of the music follows a mapping where increasing sensor values gradually lead to more complex and louder music (Fig. 1). The final stage of music is tailored to an AS amplitude that is considerably greater than in normal walking (Video 1).

**Video 1:** This is a patient with Parkinson's disease demonstrating the CuraSwing technology. When walking away from the camera with his habitual walking pattern the reduced swing of the left arm only elicits a weak musical signal (with basic instrumentation). When walking toward the camera he intentionally starts to swing the left arm. The increase of arm movement is captured by the smartphone sensors and elicits a more powerful signal (i.e. more complex and louder music).

The app enables 4 different musical tempi (106, 112, 118, and 125 bpm). It was set to match the cadence of the second test walk (focused) closely. The integer values of the APDM software were rounded off as follows: ≤108 steps/min to 106 bpm; ≥109 steps/min and ≤114 steps/min to 112 bpm; ≥ 115 steps/min and ≤121 steps/min to 118 bpm; and ≥122 steps/min to 125 bpm. Between the walks, patients had a 5‐min rest. Before the third walk, subjects familiarized themselves with walking with the music feedback for 3 min and were informed that greater arm swing will lead to a more complex and louder music. For the following third test walk, they were again asked to swing the more affected arm as much as possible and to walk roughly at the tempo of the music. Synchronizing steps with the musical beat was not required explicitly to enable pre‐conscious audio‐motor entrainment.

Healthy subjects (HS) performed 4 test walks over 40 m in a straight line at comfortable, slow, fast, and again comfortable walking speed, respectively. Data from the last 3 walks were used for analysis.

The statistical analysis was carried out with R (version 4.0.3) and MATLAB (Release 2016a) (The MathWorks, Natick, MA). To check for differences between the 3 walking modi, linear mixed effects models were run with the R package NLME using walking modus (fixed effect factor) and random effect intercept. For the parameters arm velocity and ROM, the second fixed effect factor PD‐side and the corresponding interaction term were added to the model. Main effects were checked by using the ANOVA function. If significant main effects were found, post hoc comparisons were carried out with R package emmeans, adjusting the *P* values using the procedure of Benjamini and Hochberg to control for the false discovery rate under control. In the case that standardized residuals were not normally distributed (checked by Shapiro Wilk's test), a Friedman test was performed instead. The post hoc analysis was then carried out using the Wilcoxon test. This was only necessary for the ROM of sternum rotation.

A linear regression analysis was done with MATLAB for the relation of gait velocity and AS ROM values (left and right) in the HS. Confidence limits were calculated for the lowest to the highest observed velocity.

## Results

We evaluated a sample of 30 patients with PD and compared AS and other gait kinematics to 32 age‐matched HS (Table 1). Two patients were de novo, all others received levodopa (l‐dopa) with or without additional administration of dopamine receptor agonists (n = 18) (Table 1).

**TABLE 1:** Subject data of individuals with PD and HS

	PD	HS
Number	30	32
Age (y)	62.1 ± 7.0	64.5 ± 9.0
Gender (F/M)	12/18	17/15
Height (cm)	173.1 ± 10.0	172.1 ± 10.3
Weight (kg)	81.6 ± 14.5	78.8 ± 13.9
Disease duration (y)	4.6 ± 3.8	N/A
Hoehn & Yahr stage	2.0 ± 0.6	N/A
UPDRS III motor score	21.1 ± 8.8	N/A
Levodopa equivalent daily dose (mg)	738.6 ± 426.0	N/A

Abbreviations: PD, Parkinson's disease; HS, healthy subjects; F, female; M, male, UPDRS, Unified Parkinson Disease Rating Scale.

In PD subjects, the primary outcome measures AS ROM and PAV for the right and left arms increased for the modus focused walk when compared to baseline (*P* < 0.001). Walking with CuraSwing led to further improvements in both measures (*P* < 0.001) (Table 2). The AS ROM increase from baseline to CuraSwing modus was greater on the more affected arm where the feedback device was worn when compared to the increase on the less affected side (*P* = 0.03). The respective ndASI and regularity values improved from baseline to focused walking (*P* < 0.001). Here, CuraSwing did not lead to an additional improvement (Table 2). All patients showed an increase of AS in the focused modus compared to baseline. A further increase with CuraSwing was seen in 29 of the 30 participants. The cadence did not change significantly from baseline to focused walking and rose slightly from focused walking to CuraSwing modus (*P* = 0.04), whereas stride length increased strongly over all 3 conditions (*P* < 0.001). The ROM values of the obliquity of the 5LV increased significantly over all 3 modi (*P* < 0.001). Accordingly, the *P* values calculated for the rotation of the sternum increased in favor of CuraSwing (*P* < 0.001). The stride time variability did not differ between the 3 conditions (Table 2).

**TABLE 2:** Kinematic measures and regularity (a measure of similarity of consecutive arm swings) (mean ± standard deviation) for 30 PD patients

	Baseline		Focused		CuraSwing	
	More affected side	Less affected side	More affected side	Less affected side	More affected side	Less affected side
Arm swing ROM (°)	13.5 ± 5.3	36.3 ± 17.7	53.2 ± 20.1^*^	68.8 ± 22.0^*^	75.5 ± 23.0^*^ ^,^ ^**^	85.0 ± 29.0^*^ ^,^ ^**^
Arm swing peak angular velocity (°/s)	55.5 ± 17.8	127.5 ± 56.6	176.6 ± 66.1^*^	233.0 ± 73.6^*^	248.5 ± 77.4^*^ ^,^ ^**^	286.6 ± 95.6^*^ ^,^ ^**^
Regularity (0–1)	0.909 ± 0.045	0.951 ± 0.027	0.962 ± 0.015^*^	0.967 ± 0.015^*^	0.966 ± 0.017^*^	0.965 ± 0.024^*^
Cadence (spm)	111.6 ± 6.4		111.9 ± 6.1		112.9 ± 6.1^*^ ^,^ ^**^	
Gait velocity (m/s)	1.16 ± 0.10		1.23 ± 0.12^*^		1.29 ± 0.13^*^ ^,^ ^**^	
Stride (m)	1.25 ± 0.10		1.32 ± 0.12^*^		1.37 ± 0.13^*^ ^,^ ^**^	
Stride time variability (%)	1.69 ± 0.53		1.61 ± 0.61		1.50 ± 0.63	
Obliquity 5th vertebral joint (°)	8.6 ± 2.7		10.7 ± 3.3^*^		12.1 ± 3.5^*^ ^,^ ^**^	
Rotation sternum (°)	3.6 ± 0.9		4.1 ± 1.0^*^		4.7 ± 1.3^*^ ^,^ ^**^	

* Significant difference to baseline.

** Significant difference to focused.

The linear regression of the relation between gait speed and AS ROM values (right and left sides) of the HS revealed that higher velocities were associated with higher AS ROM values.

**FIG. 2:**
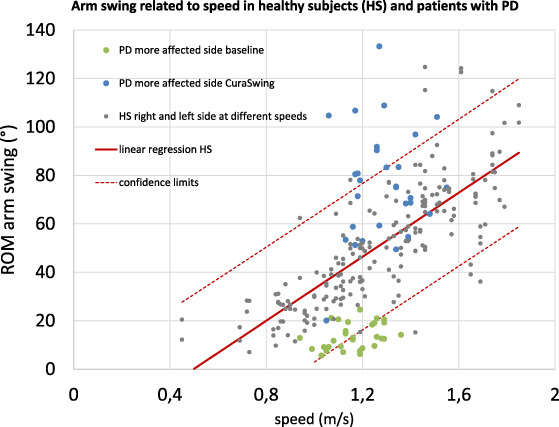
Relation of arm swing and gait velocity for patients at baseline and with CuraSwing and for healthy subjects at various tempi.

When plotting the AS ROM values of PD subjects with the linear regression of HS, 16 of 30 PD subjects performed outside the confidence limits at baseline. With the use of CuraSwing PD, subjects increased their AS and reached normal ROM values and beyond (Fig. 2).

## Discussion

Human or technical feedback and motivational stimulation (feedforward) provide efficient means to improve movement amplitude in patients with PD.^20^, ^29^, ^30^, ^31^ Musification as provided by the novel smartphone application CuraSwing conveys auditory bio‐feedback of movement execution and aims to maintain the attentional focus.^23^ In addition, the generated music serves as a rhythmic auditory cue that optimizes motor control via audio‐motor synchronization.^11^ In CuraSwing, the music becomes richer and louder when the movements are faster and larger, therefore, simultaneously providing rewarding feedback and stimulating cueing.

In the present study, increasing amplitudes of AS on the more affected side were associated with improvement of contralateral AS, trunk‐rotation, and leg movements during gait in patients with PD. Patients were able to improve performance by attentional effort. However, maximal improvements of AS and gait parameters were obtained when using CuraSwing.

The voluntary increase of AS leads to improvement of gait initiation^32^ and kinematics.^33^ When instructed to walk while deliberately swinging the arms, both shoulder and elbow excursions increased (118% and 197%, respectively), walking velocity increased by 20%, and step length increased by 18% in a study by Behrman and colleagues.^33^ In the present study, voluntary increase of AS measured on the more affected side was even higher (+338.9% for focused vs. baseline walking) and was significantly exceeded by musification of AS acceleration (+529.5% compared to baseline). The AS improvements occurred on both sides of the body as confirmed by ndASI values. Plate et al^34^ had suggested an asymmetry index of >50 to be pathological. In our patient sample, the asymmetry indices improved from baseline to focused walking and reached a physiological level. Symmetry of AS was also observed in the CuraSwing condition despite the subjects' increased attention to the more affected arm. Musical feedback for 1 arm can, therefore, lead to the improvement of bilateral AS. Larger amplitudes were accompanied by an increased regularity of AS suggesting improved motor control.

The music of CuraSwing is metrically structured and can be adjusted to the preferred stepping frequency (cadence) of the patient. In contrast to RAS, where higher cadences are used to obtain faster gait,^11^ the CuraSwing paradigm aims to achieve a physiological gait pattern by improving movement amplitudes without increasing cadence. This concept resulted from observations made in preliminary clinical experience where musification at increased tempos did not lead to higher increases of AS. Improvement of stride length (+3.4%) in the CuraSwing condition clearly exceeded the small increase in cadence (+0.94%) when compared to focused walking. This shows that the effect of musification on gait velocity is not mediated by quicker stepping, but by improving the amplitude of leg movements. The small increase in cadence might be attributed to the rounding off of the algorithm in setting the musical tempo and to the motivational effect of the music. Stride time variability, which is associated with postural instability and risk of falling,^35^ was low at baseline and did not increase with the experimental conditions.

Comparison with AS in HS showed that patients were able to achieve AS amplitudes within or beyond the normal range when being provided with musification feedback. Supra‐normal amplitudes of AS are not needed for habitual walking but are used in exercise interventions such as LSVT‐BIG to enhance awareness for movements and to achieve normal amplitudes instead of hypokinesia in daily routines.^29^, ^31^ Patients with PD are able to increase movement amplitudes temporarily by voluntarily focusing on performance. However, this effect rapidly diminishes when attention is distracted, possibly because of disturbances in sensorimotor processing and movement perception^31^, ^36^ including problems in sensory attenuation.^37^ The additional effects of acoustic movement feedback and audio‐motor synchronization with CuraSwing were shown to exceed the immediate effect of attentional focusing. Moreover, this type of feedback can be provided continuously without time limitations. The continuous provision of feedback during a motor task, along with rhythmic auditory and emotional stimulation^11^, ^13^ with CuraSwing, can be used to achieve a sustained increase of AS to a normal level in habitual walking or to enhance exercise with supra‐normal amplitudes.

Our approach extends the spectrum of options for the use of music as a component for gait training in patients with PD. It has been stated before that music has the potential to improve training and learning processes in PD by the simultaneous engagement of different neural systems^38^ and by bypassing disturbed motor circuits in the brain.^39^ In patients with PD, music can have a powerful effect on hypokinesia^40^ and directly influence motor symptoms on a short term time scale.^41^ Sustaining enlarged AS amplitude requires a high level of attention in patients with PD. It would be interesting to evaluate whether musification would also induce enhanced AS when attention is not volitionally focused on this task (e.g. in dual‐task situations).

As a therapeutic aid, music is easily available and can stimulate sensorimotor processing without requiring attention.^42^ Making music with one's own movement has been shown to reduce the perceived exertion and pain during strenuous training.^43^, ^44^ Easy accessibility, intuitive usability, immediate efficiency, pleasant entertainment, and effortless application are features of movement musification that help to overcome lack of motivation as a frequent obstacle for continuous domestic training for patients with PD.^45^ The effects of CuraSwing on spatial gait parameters suggest that musification could also be effective in other therapeutic areas where feedback and stimulation of movement amplitudes are required. The CuraSwing technology could, therefore, be a complementary element for other motivational smart phone applications that have recently been introduced as promising means to enhance domestic exercise for patients with PD.^46^

A limitation of our study is the lack of follow‐up precluding the evaluation of carry‐over effects of AS‐musification into habitual walking without musical feedback. Because of the fixed order of experimental conditions, training effects could be a concern. Yet, we did not observe increases of movement amplitude in the course of the 320 m walks, but abrupt changes when switching from one condition to the next. We did not assess the subjective perspective of patients in this study. Future studies on CuraSwing should include qualitative analyses of patients' experience and the consequences for motor performance. Most patients in the present study were mildly to moderately affected by PD. Because gait disorders become more pronounced in advanced stages of PD, future studies on CuraSwing should assess safety and effectiveness in more severely impaired PD patients.

In conclusion, stimulating musical feedback of AS with the CuraSwing application was shown to improve gait kinematics in patients with PD. Further studies are needed to evaluate long‐term transfer of improvements into habitual walking. Additionally, the comparison with other established exercise techniques to improve gait is warranted. When considering the role of attention, the CuraSwing application might also be tested in a dual task approach where arm swing is not the (main) therapeutic target. The combination of movement musification with other training approaches to counteract hypokinesia is a tentative perspective.

## Author Roles

(1) Research project: A. Conception, B. Organization, C. Execution; (2) Statistical Analysis: A. Design, B. Execution, C. Review and Critique; (3) Manuscript Preparation: A. Writing of the First Draft, B. Review and Critique.

S.M.: 1A, 1B, 1C, 2A, 2C, 3A, 3B

A.S.: 2A, 2B, 2C, 3B

E.W.: 1C, 3B

F.G.: 1B, 3B

W.M.: 3B

G.E.: 1A, 1B, 2A, 2C, 3B

## Disclosures

### Ethical Compliance Statement

The study protocol was approved by the ethics committee of the medical board Brandenburg (AS 13(bB)/2020). Study subjects gave their informed written consent according to the Helsinki declaration. We confirm that we have read the Journal's position on issues involved in ethical publication and affirm that this work is consistent with those guidelines.

### Funding Sources and Conflicts of Interest

This study was funded by Deutsche Parkinsonvereinigung, Neuss, Germany. The authors declare that there are no conflicts of interest relevant to this work.

### Financial Disclosures for the Previous 12 Months

G.E. received honoraria for advisory board or consultancy from AbbVie Pharma, BIAL Pharma, Biogen, Desitin Pharma, STADA Pharma, and Neuroderm. He received speaker honoraria from AbbVie Pharma, BIAL Pharma, Britannia Pharma, Desitin Pharma, Licher, UCB Pharma, and Zambon Pharma. G.E. received royalties from Kohlhammer Verlag and Thieme Verlag. F.G. received honoraria from AbbVie Pharma, Merz Pharma, and BIAL Pharma. W.M. receives or received funding from the European Union (PI of KEEP CONTROL, Co‐PI of IDEA‐FAST), the German Federal Ministry of Education of Research, German Research Council, The Michael J. Fox Foundation, Robert Bosch Foundation, Neuroalliance, Lundbeck, Sivantos, and Janssen. He received speaker honoraria from AbbVie, Bayer, GlaxoSmithKline, Licher MT, Neuro‐Kolleg Online‐Live, Rölke Pharma, Takeda, and UCB; was invited to advisory boards of AbbVie, Biogen, Kyowa Kirin, Lundbeck, and Market Access and Pricing Strategy, is an advisory board member of the Critical Path for Parkinson's Consortium; and an editorial board member of Geriatric Care. W.M. serves as the co‐chair of the MDS Technology Task Force. The authors declare that there are no additional disclosures to report.
